# A systematic review of the “promising zone” design

**DOI:** 10.1186/s13063-020-04931-w

**Published:** 2020-12-04

**Authors:** Julia M. Edwards, Stephen J. Walters, Cornelia Kunz, Steven A. Julious

**Affiliations:** 1 School of Health and Related Research, The University of Sheffield, Sheffield, UK; 2 Boehringer Ingelheim, Biberach an der Riss, Biberach, Germany

**Keywords:** Promising zone, Unblinded sample size re-estimation, Systematic review, Sample size calculations

## Abstract

**Introduction:**

Sample size calculations require assumptions regarding treatment response and variability. Incorrect assumptions can result in under- or overpowered trials, posing ethical concerns. Sample size re-estimation (SSR) methods investigate the validity of these assumptions and increase the sample size if necessary. The “promising zone” (Mehta and Pocock, Stat Med 30:3267–3284, 2011) concept is appealing to researchers for its design simplicity. However, it is still relatively new in the application and has been a source of controversy.

**Objectives:**

This research aims to synthesise current approaches and practical implementation of the promising zone design.

**Methods:**

This systematic review comprehensively identifies the reporting of methodological research and of clinical trials using promising zone. Databases were searched according to a pre-specified search strategy, and pearl growing techniques implemented.

**Results:**

The combined search methods resulted in 270 unique records identified; 171 were included in the review, of which 30 were trials. The median time to the interim analysis was 60% of the original target sample size (IQR 41–73%). Of the 15 completed trials, 7 increased their sample size. Only 21 studies reported the maximum sample size that would be considered, for which the median increase was 50% (IQR 35–100%).

**Conclusions:**

Promising zone is being implemented in a range of trials worldwide, albeit in low numbers. Identifying trials using promising zone was difficult due to the lack of reporting of SSR methodology. Even when SSR methodology was reported, some had key interim analysis details missing, and only eight papers provided promising zone ranges.

## Introduction

Sample size calculations performed prior to the start of a trial require assumptions about the hypothesised treatment response and variability. Incorrect assumptions can result in a trial being either underpowered or overpowered. Both situations pose ethical concerns: either recruiting patients to an investigational treatment without being able to fully answer the research question or subjecting more patients than necessary to a potentially inferior treatment when there is sufficient information already available to answer the research question [1].

The CONSORT Statement in 2010 highlighted the prevalence of underpowered trials in practice, stating “Reviews of published trials have consistently found that a high proportion of trials have low power to detect clinically meaningful treatment effects” [2]. Methods have been developed whereby trial assumptions may be investigated during the progression of a trial, and sample size may be increased if necessary in order to maintain power.

Blinded methods that do not require knowledge of the treatment group allocation of patients are generally widely accepted by regulatory agencies [3]. However, unblinding treatment allocation can introduce bias and can inflate type I error [4].

In 2011, Mehta and Pocock introduced the “promising zone design”, building on work by Chen et al. in 2004 and making it more user-friendly for easier implementation [5, 6]. At the interim analysis, conditional power is calculated based on observed data and assuming the future treatment effect will follow the current trend observed so far.

- At the analysis, the conditional power value will fall in one of the three pre-specified “zones”: “unfavourable” (i.e. not worth the increase in sample size required to maintain originally planned power).
- “Promising” (treatment effect may be lower than expected, but not so low that conditional power cannot be recovered through increasing the sample size).
- “Favourable” (the treatment effect is “sufficiently favourable”, and sample size does not need to increase to ensure power is maintained).

There has been much discussion regarding the implementation of the promising zone design and with arguments that the design is not optimal due to using a conventional test statistic in the final analysis [7]. However, the simplicity in the design has an appeal to many researchers wishing to implement an unblinded sample size re-estimation (SSR).

### Motivation for systematic review

Promising zone is still relatively new in its application, and the extent of its uptake in practice is currently unknown. The concept was adapted from previous research to make the methodology known at the time more accessible to researchers to implement in trials. There are however certain factors for trialists to consider before implementing a SSR, which will vary from trial to trial, such as time to primary outcome, restricted increase in sample size or stopping boundaries. As no clear rules exist for such considerations, trials implementing this methodology may differ substantially. Therefore, it would be useful to get an indication of the situations in which trialists are choosing to implement this methodology, and what key considerations are being used.

The aim of this paper is to investigate and synthesise the current viewpoints of the promising zone design and its implementation in current trials by systematically reviewing the literature.

## Search strategy

Three distinct methods were used in order to comprehensively cover a wide range of literature: database searching, pearl growing and grey literature searching.

A preliminary database search was conducted in order to extensively investigate the terminology used. In light of the preliminary research, three search strategies were implemented individually and then combined:

1. “sample size re-estimation” OR “sample size reestimation” OR “sample size adjustment” OR “sample size readjustment” OR “sample size modification” OR “sample size recalculation” OR “sample size reassessment” OR “*creased sample size” OR “*crease in sample size” OR “adaptive sample size”
2. “promising zone” OR “promising region”
3. “promising” AND “results” AND “conditional power”

Final Search: (1) AND (2 OR 3)

Online databases searched included PubMed, Web of Science (WoS), Cochrane Database, CINAHL and OVID (Including MEDLINE and PsychINFO). Additionally, ClinicalTrials.gov was searched for search strategy (1). Additional trial documents from trials identified through ClinicalTrials.gov were searched for any indication of promising zone usage.

Pearl growing techniques were implemented using the Promising Zone paper published by Mehta and Pocock [5]. Web of Science and Google Scholar were searched for publications citing this work. Additionally, all publications included in the review were searched for additional records (e.g. trials written as case studies in an included publication were included).

FDA, EMA, NICE, PSI and Cytel websites were all searched for any additional documentation or resources related to promising zone, including conference abstracts, presentations or guidance.

### Restrictions

The database search included all publications up to 31 August 2020, and is restricted to publications or translations of publications being available in the English language, and must be accessible. Furthermore, publications must refer to “promising zone” in > 1 sentence to be included in the methodology systematic review. This ensures the exclusion of publications only referring to promising zone as an option for SSR but offers no further information. Additional trial-specific inclusion criteria included randomised controlled studies only and must identify promising zone as the SSR method. Trials were excluded if the SSR method was unknown.

### Search results

**Fig. 1:**
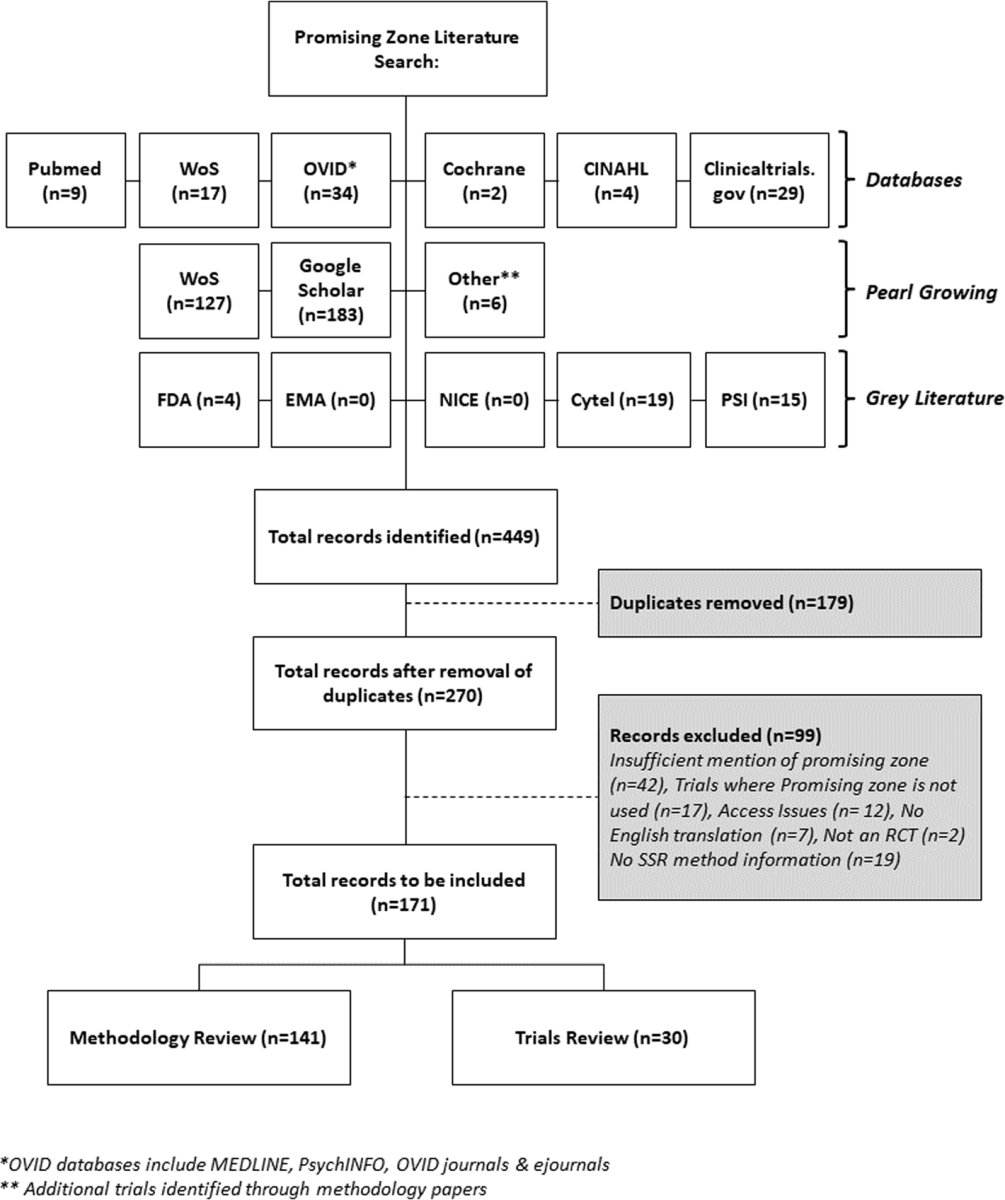
Flowchart of the literature search, detailing numbers included in the review and reasons for exclusion

A flowchart to describe the information search is shown in Fig. 1. Six databases were searched, identifying a total of 95 records. Google Scholar found 183 records citing Mehta and Pocock’s promising zone publication, and Web of Science found 127. A further 6 case studies were mentioned in records already identified and were included for screening. Finally, an additional 38 grey literature items were identified for screening for inclusion. The three search methods combined gave a total of 449 records. After removing 179 duplicate records, 270 unique records were considered for inclusion. A total of 99 records were excluded for the following reasons: insufficient mention of promising zone (*n* = 42), trials that did not use promising zone (*n* = 17), unable to access (*n* = 12), unavailable in English (*n* = 7), not a randomised controlled trial (*n* = 2) and not clear whether promising zone was used as the SSR method (*n* = 19).

A total of 171 records were included in the systematic review: 141 of which were methodological papers, and 30 were trials.

## Methodological review

### Background

In 2004, Chen et al. showed that, despite a SSR, a conventional hypothesis test could be carried out without inflating the type I error, provided a sample size increase only occurs if conditional power exceeds 50% [6]. In 2008, Gao et al. extended the range in which a researcher could increase the sample size in a K-stage group sequential design (GSD) and still use a conventional final analysis without inflating type I error [8]. Mehta and Pocock made this research more accessible to those wishing to implement a SSR [5]. Their methodology includes the derivation of the promising zone, the formula for conditional power and the calculation for the second-stage sample size required. Furthermore, case studies are presented to illustrate the potential uses for SSR using this method. Since 2011, there has been much discussion regarding the promising zone methodology, and many have criticised for a number of reasons. Both strengths and limitations are synthesised from the publications from the systematic review.

### Strengths

The CONSORT Statement in 2010 [2] highlights the prevalence of underpowered studies in publicly funded trials. This has underlying implications for research, as too few subjects being recruited may not fully answer the research question being addressed and can be susceptible to making false-negative conclusions due to its low statistical power [9]. One of the key benefits of using promising zone is that it reduces the risk of an underpowered trial [10]. Additionally, Mehta advocates the use of an unadjusted analysis, making the methodology approachable to researchers and allows for easy implementation and interpretation.

Type I error inflation is minimal, even when the algorithm triggers as an increase in sample size [11], corresponding to the work by Broberg [12], who shows any inflation of type I error using promising zone methodology is low/nominal.

Promising zone designs can also greatly increase gains in conditional power as opposed to a GSD or fixed sample size design, particularly for a promising treatment effect, or a delayed treatment effect in time-to-event trials [13]. This however has no utility in reality unless this also corresponds to a gain in unconditional power [13].

This design is particularly useful for small biotech/medical device companies who may not be able to invest a large amount of funds or other resources to large trials [14]. Instead, they can commit resources in stages [15] and are more likely to obtain the funding required if the treatment effect can be shown to be promising [16].

A simulation study investigating a seamless phase II/III trial with promising zone design suggested some appealing advantages over classical designs including time, cost and sample size savings, which could speed up the development of biosimilars [17]. Additionally, it has been suggested that promising zone designs could be particularly useful in early-stage exploratory studies where very little is known about the treatment effect [18], and in clinical trials of rare diseases [19]. However, more research is required to fully investigate the benefits in both of these areas.

Finally, using an unadjusted final analysis has the attractive property that all patients are equally weighted in the analysis, unlike weighted test statistics. This has been referred to as the “one patient-one vote” principle [20].

### Limitations

The conditional power calculation relies on a future treatment effect assumption, which Mehta and Pocock use the current trend of observed data rather than that used in the planning stage. Glimm suggests that using the current trend assumption yields an unstable estimate of the true conditional power, which is highly variable early on in the trial, and is therefore unwise to base a SSR on this estimate [7]. This comes from effectively using the estimated treatment effect (${\hat{\delta}}_1$) twice, meaning that any random deviation of ${\hat{\delta}}_1$ from its true value (*δ*_1_) could consequently impact conditional power considerably [7]. Mehta and Pocock realise this however and note that “The point, however, is not to estimate δ with precision but rather to identify if the interim results are promising. The conditional power estimate is one way of accomplishing this.”

A key concern in recent literature is the conservative nature from using an unadjusted critical value in the final analysis [21–25] and therefore is inefficient. The main argument for this approach from Mehta is the ease of understanding and implementation [20]. However, it has been highlighted that there are a number of other studies with non-standard final analyses that are widely accepted and understood, such as GSDs [26].

An added constraint to the algorithm is that sample size may never decrease below the originally planned sample size. Hung et al. showed results falling in the promising zone will have much higher conditional power than required, and decreasing sample size to maintain the minimal conditional power would be advantageous to promising zone [27, 28]. Mehta responded, pointing out that FDA guidelines clearly state that an adaptive SSR should only be used for increases in sample size, and therefore, their constraint is to enable these guidelines [4, 14].

The promising zone should be pre-specified in advance of the trial and must be strictly adhered to. Regulatory concerns regarding compliance have been raised, as any deviation could result in type I error inflation [29]. Additionally, Mehta and Pocock give CP_min_ values (the smallest value of conditional power in which results could be considered “promising”). Prior to the start of the trial, researchers may increase this value if they wish and thus decrease the promising zone. However, decreasing this zone can decrease the trial’s overall power [5].

Although useful for small biotech companies, there is an opportunity for this methodology to be misused. Turnbull suggests that a company unable to afford a full trial may plan an overly optimistic treatment effect with SSR design and lure investors to fund an increased trial when a smaller treatment effect is unsurprisingly found [30]. The risk however is that the treatment effect is too optimistic, falling in the unfavourable zone and leads to an underpowered trial.

Finally, Jennison and Turnbull have presented findings that the greatest gain in power per patient actually lies outside the promising zone [31].

### Considerations

Whilst there is no fixed recommendation of interim analysis timing within the literature, there is a general consensus that later is better. Liu and Lim advise against an early interim analysis, as the treatment effect is unstable early on in the trial [32]. This lowers the ability to rescue an underpowered trial and thus retracts any benefit from using a promising zone design. Both Levin et al. [33] and Gaffney and Ware [34] agree that the later the interim analysis, or closer to the minimal sample size, the more efficient the design becomes. However, there are also logistical considerations, as deciding close to the minimal sample size may delay the continued recruitment whilst any adaptations are being implemented [35].

Additionally, the length of time between randomisation and primary outcome data becoming available for patients should be considered, as this will impact the amount of data available at the interim analysis. Jennison and Turnbull highlight the benefit that promising zone designs have over GSDs when the time to outcome is long [31]. However, Hampson and Jennison have since developed methodology for GSDs to better deal with these pipeline patients [36]. Furthermore, accrual periods play a role on the utility of promising zone designs. If it is expected that a large number of patients, or indeed all, are to be recruited whilst the interim analysis takes place, then the impact of any decision will be minimal. Whilst a sample size increase may take place after recruitment had initially finished, logistical restrictions such as treatment supply may need to be overcome which may delay continued recruitment.

The extension of the promising zone range in Mehta and Pocock’s work [5], compared to that of Chen et al. [6], leading to a larger range where sample size should be increased. Due to the decreasing nature of the function used to determine second-stage sample size, a larger increase in sample size may be required. When doubling or tripling the sample size, Chen et al. have said that this extension can be useful [37]. However, there may be many instances where even doubling of the sample size would be infeasible. An appealing solution is the incorporation of a maximum sample size cap, *n*_max_, in which the sample size increase cannot exceed.

For example, when looking at a portfolio of studies, it is inefficient to extend past a certain number of subjects as only small increases in power can be seen past this point [38]. Similarly, Gaffney and Ware found that introducing a maximum sample size restriction can restrict the increase in power, stating that the smaller the maximum cap of *n*, the smaller the expected increase in power [34]. Gaffney and Ware also show the intuitive result whereby the larger the cap, the larger the expected gains in expected power increase and the expected sample size. However, increasing the maximum cap from 1.5 times to 2.5 times, the original planned sample size reduced the efficiency of SSR (defined as the expected power of the adaptive design minus the power of the fixed sample size design) unless the true effect size is small [34]. Liu et al. found that if a conservative promising zone range was used, and sample size is restricted to no more than a 50% increase, then there is no loss in efficiency [39]. Furthermore, any loss in power is counteracted by the substantial gain in conditional power when the treatment effect is found to be promising [39].

The value of *n*_max_ must be pre-specified and kept constant throughout the trial, as type I error can be inflated if this value is lowered at any point after the start of the trial [40], with the promising zone lower boundary being based on the original maximum restriction in sample size. In practice, the realistic magnitude of *n*_max_ is dependent on logistical restraints such as funding, resources available or available patients. Furthermore, publicly funded and industry trials may have different concepts of a maximum feasible sample size.

### Extensions

Methodology has been proposed in order to increase the efficiency of SSR, as alternatives to promising zone. Jennison and Turnbull present methods using an inverse combination test as opposed to the conventional analysis, allowing the sample size to be increased at any value of ${\hat{\delta}}_1$ without inflating the type I error [31]. They also optimise the increase in sample size in terms of expected sample size. Additionally, a reduction in sample size is also considered but is constrained to a minimum sample size level [14]. This methodology opens up the broad framework proposed by Mehta and Pocock for use in future clinical trials.

Additionally, Bowden and Mander implement a “reverse Li, Shih and Wang method” [22] based on the work of Li et al. [41]. This includes an algorithm, identifying a number of designs consistent with the significance level and critical values. From this, the minimum sample size can be chosen such that unconditional power equals 1-β.

## Trials review

Trial characteristics from the 30 studies found in the systematic review are summarised in Table 1. Where information was unobtainable from the study documents available, trialists were contacted for more details.

**Table 1:** Trial characteristics

	Trials included in the review (***N*** = 30)	
	***n***/***N***	%
**Interim analysis details**		
Number of interim analyses planned		
1	28/30	93.3
2	2/30	6.7
Timing of the first interim analysis^1^ (% of initially planned sample size)		
Median (IQR)	60.0	(41.1, 72.6)
Status of information at the first interim analysis		
Completed data collection (primary outcome)	14/23	60.9
Recruited	5/23	21.7
Events occurred	4/23	17.4
Result at first interim analysis		
Continue	4/13	30.8
Stop	2/13	15.4
SSR	7/13	53.8
Maximum sample size percentage increase^2^		
Median (IQR)	50.0	(35.3, 100.0)
Actual sample size percentage increase^3^		
Median (IQR)	11.56	(1.12, 42.3)
Stopping boundaries?^4^		
Yes	15/21	71.4
If yes, type of stopping boundary		
Efficacy	4/15	26.7
Futility	5/15	33.3
Efficacy and futility	4/15	26.7
Not specified	2/15	13.3
**General trial characteristics**		
Primary outcome type		
Binary	16/33	48.5
Continuous	10/33	30.3
Time-to-event	7/33	21.2
Time to primary outcome		
0–3 days	5/30	16.7
1–6 weeks	6/30	20.0
8–12 weeks	6/30	20.0
5–6 months	4/30	13.3
1–5 years	9/30	30.0
Number of groups		
2	29/30	96.7
4	1/30	3.3
Study design		
Cluster	1/30	3.3
Crossover	1/30	3.3
Parallel group	28/30	93.3
Phase^6^		
I	1/20	5.0
II	6/20	30.0
III	13/20	65.0
Disease area		
Cardiovascular	5/30	16.7
Diabetes	4/30	13.3
Neurology	7/30	23.3
Oncology	5/30	16.7
GI	2/30	6.7
Others****	7/30	23.3
Progress		
In progress	12/30	40.0
In analysis	3/30	10.0
Completed	15/30	50.0
Funder type		
Industry	17/30	56.7
Non-industry	13/30	43.3

^1^No timing of interim look data from 3 trials

^2^No maximum sample size reported for 9 trials

^3^Based on the 12 trials that implemented a SSRE

^4^No information on usage of stopping boundaries for 9 trials

^5^Three trials had > 1 primary outcome: continuous and binary (*n* = 1), binary and time-to-event (*n* = 1) and two time-to-event (*n* = 1)

^6^Other disease areas include one trial each in critical care, emergency medicine, haematology, dental care, orthopaedic, ostomy and pain

Twelve trials (40%) are currently still in progress, and three (10%) are in the analysis stage. Despite no result publications, all 10 had sufficient information to be included in this review. Almost all trials included just one interim analysis in which a sample size reassessment could be performed (28/30; 93%). The median timing of the first interim analysis was over halfway through the study (60.0% through the initial sample size recruited; IQR 41%, 73%), calculated as a percentage of the originally planned sample size. Three studies were excluded due to insufficient information being reported for this calculation. All 30 trials (100%) reported planning for an interim analysis prior to the start of the trial. Of the 13 trials that have been completed and reported interim analysis-specific details, 4 trials (31%) continued to the originally estimated sample size, 2 trials (15%) terminated early (one for efficacy, one for futility) and 7 (54%) increased their sample size according to conditional power calculations as indicated by the promising zone methodology. Fifteen trials (50%) used stopping boundaries in addition to the SSR.

Only 21 of the 30 studies (70%) reported a maximum sample size increase the investigators were willing to consider, and the median actual increase as a percentage of the original sample size was 11.6% (IQR 1.1%, 42.3%). Three trials had more than 1 primary outcome (details in footnote number 5), resulting in a total of 33 individual primary outcomes from the 30 trials. Half of the outcomes were binary (16/33; 49%). Data for 5 (17%) primary outcomes could be collected within 3 days, whereas the 9 (30%) time-to-event primary outcomes took between 1 and 5 years for primary outcome data to become available.

One study had a crossover design with 4 treatment groups. This study incorporated a group sequential approach, recruiting the initial sample size as cohort 1, and only recruiting a second cohort based on the findings at the interim analysis. Due to the nature of emergency medicine studies, one trial uses a cluster design, randomising additional clusters and therefore additional patients if necessary.

Of the 20 trials that reported a study phase, two thirds were phase III. There were a range of disease areas investigated in the trials, including neurology, oncology, cardiovascular, diabetes, gastrointestinal, critical care, haematology, dental care, emergency medicine, orthopaedic, ostomy and pain. Over half of the trials were funded by industry (17/30; 57%). Interestingly, one study used an adaptive switch design, where a trial can start as a non-inferiority trial, and based on an interim analysis, it may remain as non-inferiority, switch to a superiority endpoint or switch to an adaptive design using promising zone. However, this trial terminated early as non-inferiority could not be shown.

Two of the trials did not use the sample size rule recommended by Mehta and Pocock. Instead, if the results fell in the promising zone, the sample size was increased by a pre-specified amount, regardless of the observed value of conditional power within the promising zone, known as a stepwise design.

Promising zone boundaries used for 19 trials are reported in Table 2. Five of these trials also reported a “four-zone” trial based on observed conditional power values: one trial stopped and claimed non-inferiority if CP < 0.2; three trials stopped for futility for values CP < 0.03, CP < 0.1 and CP < 0.2; and one trial included an enrichment design based on a subgroup of patients.

**Table 2:** Conditional power ranges of the promising zone

CP lower boundary	CP upper boundary
0.3	0.95
0.3	0.9
0.3	0.9
0.3	0.8
0.36	0.8
0.38	0.8
0.385*	0.8
0.385*	0.8
0.385*	0.8
0.385*	0.8
0.385*	0.8
0.41	0.8
0.43	0.85
0.5	0.9
0.5	0.8
0.5	0.8
0.5	0.8
0.708	0.8
(Missing)	0.8

Conditional power lower and upper boundaries of the promising zone for the 15 trials that provided conditional power ranges for the promising zone

*Five trials from one centre all used the same CP range for the promising zone

The primary reasons for using sample size re-estimation methodology are presented in Table 3.

**Table 3:** Reasons for using sample size re-estimation methodology

Reason	Number
Uncertainty in treatment estimates	*n* = 9
Simplicity/convenience	*n* = 4
Efficiency	*n* = 3
Ensure power is maintained	*n* = 6
Unspecified	*n* = 3

## Example of good practice

The real-time feedback, debriefing, and retraining system of cardiopulmonary resuscitation for out-of-hospital cardiac arrest trial (UMIN000021431) by Hirakawa et al. is an ongoing trial in Japan that plan to use the promising zone design. The trialists published a protocol paper outlining their initial trial, their uncertainty and clear details of the trial design they will implement in order to combat the uncertainty surrounding treatment effect estimates in order to maintain power at the end of the trial. An extract of the adaptive design methodology is given below.

In our cluster RCT, the sample size was determined using the initial guess value of odds ratio of the intervention group relative to the control group for 1-month favourable neurological survival. However, if this value is not true, the trial may not achieve the desired power. [ … ] Thus we incorporated sample size re-estimation as a form of adaptive design

Sample size re-estimation will be planned using the method proposed by Mehta and Pocock [5]. Specifically, at the time of the interim analysis, the conditional power for detecting the difference in primary outcome between two groups in the final analysis will be estimated using the accumulated data of 1500 patients [upon completion of the primary endpoint assessment]. When the conditional power is between 50% and 80%, we will increase the sample size accordingly, up to a maximum sample size of 5040. Otherwise, the trial will continue using the planned sample size.

The protocol paper clearly identifies the promising zone design including a promising range of conditional power values, the initial sample size, timing of interim analysis and maximum sample size to be considered. When reporting the design of a trial using sample size re-estimation methodology, these details would be beneficial to the development of this research.

## Discussion

Nine years following the promising zone publication, there has been a substantial amount of methodological research and comments on this design. However, there have been far fewer trials reporting the use of promising zone. It was very difficult in some cases to identify whether a trial had used SSR, and if they had, which SSR methodology had been used. For this reason, it is likely that other trials have indeed implemented this design, and maybe more prevalent than what is captured within this research. Additionally, publication bias may exist, with trials having stopped early for futility having the potential to be under-reported.

Even when trials had reported using SSR, key details regarding the design were not always reported. In fact, only 8 trials clearly defined both lower and upper promising zone limits used, without needing to contact the authors. Seven trials did not report the maximum sample size that would be considered, and one did not report the initially planned sample size. One reason for this could be due to the need for discretion in order to reduce operational bias, with sometimes not even the trial team knowing this information until the end of the study.

There was a roughly even split between industry and publicly funded trials implementing promising zone, and trials covered a broad range of therapeutic areas. The design is less common in early phase trials, with the majority being phase III. The median timing of the interim analysis was 60% through the originally planned sample size but ranged from 41 to 73%. There were a number of short-term outcomes, with some trials having primary outcome data available in a matter of days. On the other hand, this design was also used in trials considering long-term time to event endpoints, with data taking a number of years to collect.

The most common motivation reported for using promising zone design was due to the uncertainty surrounding the treatment effect. Two trials reported not using sample size re-estimation in their interim analyses despite initially planning for it, one due to lengthy follow-up (3 years) and the other due to very low recruitment.

In conclusion, despite the ongoing debates over this design, it is being implemented in practice in trials worldwide, for a wide range of situations. Trials implementing promising zone should report key interim analysis decisions where possible, to better aid the understanding and ongoing research of this design.

## Data Availability

Not applicable

## References

[1] Altman D (1980). Statistics and ethics in medical research: III how large a sample?. Bmj.

[2] Schulz K, Altman D, Moher D (2010). CONSORT 2010 Statement: updated guidelines for reporting parallel group randomised trials. BMC Med.

[3] FDA (2016). Guidance for industry: adaptive designs for medical device clinical studies.

[4] FDA (2010). Draft guidance for industry: adaptive design clinical trials for drugs and biologics.

[5] Mehta C, Pocock S (2011). Adaptive increase in sample size when interim results are promising: a practical guide with examples. Stat Med.

[6] Chen J, DeMets DL, Lan G. Increasing the sample size when the unblinded interim result is promising. Stat Med. 2004. 10.1002/sim.1688. 10.1002/sim.1688 15057876

[7] Glimm E (2012). Comments on adaptive increase in sample size when interim results are promising. Stat Med.

[8] Gao P, Ware JH, Mehta C (2008). Sample size re-estimation for adaptive sequential design in clinical trials. J Biopharm Stat.

[9] Halpern SD (2005). Adding nails to the coffin of underpowered trials. J Rheumatol.

[10] Mehta CR (2012). Designs for clinical trials.

[11] Brannath W, Gutjahr G, Bauer P (2012). Probabilistic foundation of confirmatory adaptive designs. J Am Stat Assoc.

[12] Broberg P. Sample size re-assessment leading to a raised sample size does not inflate type i error rate under mild conditions. BMC Med Res Methodol. 2013;13:94. 10.1186/1471-2288-13-94. 10.1186/1471-2288-13-94 PMC3738874 23870495

[13] Freidlin B, Korn EL. Sample size adjustment designs with time-to-event outcomes: a caution. Clin Trials. 2017. 10.1177/1740774517724746. 10.1177/1740774517724746 28795844

[14] Mehta CR (2013). Adaptive clinical trial designs with pre-specified rules for modifying the sample size: a different perspective. Stat Med.

[15] Pritchett YL (2015). Sample size re-estimation designs in confirmatory clinical trials—current state, statistical considerations, and practical guidance. Stat Biopharm Res.

[16] Posch M, Bauer P (2013). Adaptive budgets in clinical trials. Stat Biopharm Res..

[17] Uozumi R, Hamada C. Adaptive seamless design for establishing pharmacokinetic and efficacy equivalence in developing biosimilars. Ther Innov Regul Sci. 2017. 10.1177/2168479017706526. 10.1177/2168479017706526 30227103

[18] Wang SJ, James Hung HM, O’Neill R (2012). Paradigms for adaptive statistical information designs: practical experiences and strategies. Stat Med.

[19] Bayar MA, Le Teuff G, Michiels S, Sargent DJ, Le Deley MC (2016). New insights into the evaluation of randomized controlled trials for rare diseases over a long-term research horizon: a simulation study. Stat Med.

[20] Mehta CR (2016). Comments on ‘some challenges with statistical inference in adaptive designs’ by Hung, Wang, and Yang. J Biopharm Stat.

[21] Bauer P, Bretz F, Dragalin V, König F, Wassmer G (2016). Twenty-five years of confirmatory adaptive designs: opportunities and pitfalls. Stat Med.

[22] Bowden J, Mander A (2014). A review and re-interpretation of a group-sequential approach to sample size re-estimation in two-stage trials. Pharm Stat.

[23] Chen C, Anderson K, Mehrotra DV, Rubin EH, Tse A (2018). A 2-in-1 adaptive phase 2/3 design for expedited oncology drug development. Contemp Clin Trials.

[24] Tamhane AC, Wu Y, Mehta CR (2012). Adaptive extensions of a two-stage group sequential procedure for testing primary and secondary endpoints (II): sample size re-estimation. Stat Med.

[25] Bauer P, Bretz F, Dragalin V, König F, Wassmer G (2016). Authors’ response to comments. Stat Med.

[26] Shih WJ, Li G, Wang Y. Methods for flexible sample-size design in clinical trials: likelihood, weighted, dual test, and promising zone approaches. Contemp Clin Trials. 2016. 10.1016/j.cct.2015.12.007. 10.1016/j.cct.2015.12.007 26674739

[27] Hung HMJ, Wang SJ, Yang P (2014). Some challenges with statistical inference in adaptive designs. J Biopharm Stat.

[28] Hung HMJ (2016). Rejoinder to Dr. Cyrus R. Mehta. J Biopharm Stat.

[29] Hung HMJ, Wang S, Yang P, Jin K, Lawrence J (2016). Statistical challenges in a regulatory review of cardiovascular and CNS clinical trials. J Biopharm Stat..

[30] Turnbull BW (2017). Adaptive designs from a data safety monitoring board perspective: some controversies and some case studies. Clin Trials.

[31] Jennison C, Turnbull BW. Adaptive sample size modification in clinical trials: start small then ask for more? Stat Med. 2015. 10.1002/sim.6575. 10.1002/sim.6575 26172385

[32] Liu Y, Lim P (2017). Sample size increase during a survival trial when interim results are promising. Commun Stat Theory Methods.

[33] Levin GP, Emerson SC, Emerson SS (2013). Adaptive clinical trial designs with pre-specified rules for modifying the sample size: understanding efficient types of adaptation. Stat Med.

[34] Gaffney M, Ware JH (2017). An evaluation of increasing sample size based on conditional power. J Biopharm Stat.

[35] Maca J, Dragalin V, Gallo P (2014). Adaptive clinical trials: overview of phase III designs and challenges. Ther Innov Regul Sci.

[36] Hampson LV, Jennison C (2012). Group sequential tests for delayed responses (with discussion). J R Stat Soc B.

[37] Chen J, Yuan S, Li X (2018). Statistical inference following sample size adjustment based on the 50%-conditional-power principle. J Biopharm Stat.

[38] Antonijevic Z. The impact of adaptive design on portfolio optimization. Ther Innov Regul Sci. 2016. 10.1177/2168479016640020. 10.1177/2168479016640020 30231754

[39] Liu L, Hsiao S, Mehta CR. Efficiency considerations for group sequential designs with adaptive unblinded sample size re-assessment. Stat Biosci. 2017:1–15. 10.1007/s12561-017-9188-x.

[40] Wang S-J, Brannath W, Brückner M, James Hung HM, Koch A (2013). Unblinded adaptive statistical information design based on clinical endpoint or biomarker. Stat Biopharm Res.

[41] Li G, Shih WJ, Xie T, Lu J (2002). A sample size adjustment procedure for clinical trials based on conditional power. Biostatistics.

